# Modulation of adipocyte lipogenesis by octanoate: involvement of reactive oxygen species

**DOI:** 10.1186/1743-7075-3-30

**Published:** 2006-07-27

**Authors:** Wen Guo, Weisheng Xie, Jianrong Han

**Affiliations:** 1 Obesity Research Unit, Department of Medicine, Boston University School of Medicine, Boston, MA 02118, USA

## Abstract

**Background:**

Octanoate is a medium-chain fatty acid (MCFA) that is rich in milk and tropical dietary lipids. It also accounts for 70% of the fatty acids in commercial medium chain triglycerides (MCT). Use of MCT for weight control tracks back to early 1950s and is highlighted by recent clinical trials. The molecular mechanisms of the weight reduction effect remain not completely understood. The findings of significant amounts of MCFA in adipose tissue in MCT-fed animals and humans suggest a direct influence of MCFA on fat cell functions.

**Methods:**

3T3-L1 adipocytes were treated with octanoate in a high glucose culture medium supplemented with 10% fetal bovine serum and 170 nM insulin. The effects on lipogenesis, fatty acid oxidation, cellular concentration of reactive oxygen species (ROS), and the expression and activity of peroxisome proliferator receptor gamma (PPARγ) and its associated lipogenic genes were assessed. In selected experiments, long-chain fatty acid oleate, PPARγ agonist troglitazone, and antioxidant N-acetylcysteine were used in parallel. Effects of insulin, L-carnitine, and etomoxir on β-oxidation were also measured.

**Results:**

β-oxidation of octanoate was primarily independent of CPT-I. Treatment with octanoate was linked to an increase in ROS in adipocytes, a decrease in triglyceride synthesis, and reduction of lipogenic gene expression. Co-treatment with troglitazone, N-acetylcysteine, or over-expression of glutathione peroxidase largely reversed the effects of octanoate.

**Conclusion:**

These findings suggest that octanoate-mediated inactivation of PPARγ might contribute to the down regulation of lipogenic genes in adipocytes, and ROS appears to be involved as a mediator in this process.

## Background

Medium-chain fatty acids (MCFA) belong to a unique type of fatty acids that is metabolized differently from either long-chain fatty acids or carbohydrates. Dietary medium-chain triglycerides (MCT) inhibit body fat mass growth in both animals and human [1-10] Early studies suggest that this effect might be caused by rapid absorption of MCT-derived MCFA and their β-oxidation in the liver, which reduces the circulating fatty acids available to the adipocytes [11]. This model is supported by the evidence that MCFA enters the β-oxidation pathway in liver mitochondria independent of carnitine palmitoyl transferase I (CPT-I) [12]. However, it does not explain the findings that dietary MCT inhibits lipogenesis in adipocytes [13,14]. Furthermore, MCFA are recovered in the adipose tissue fatty acids up to 30 mole % in both animals and humans adapted to MCT diets [6,15-17]. These findings imply that a substantial influx of MCFA into the adipocytes occurs *in vivo*, which might affect adipose tissue function more than previously appreciated. Indeed, we found that a reduction in fat mass was associated with reduced expression of lipogenic genes and adipocyte transcription factors in MCT-fed animals [6]. This effect was reproduced in cultured adipocytes treated with octanoate [18]. When added to differentiating rodent preadipocytes, MCFA also inhibits fat accumulation and reduces expression of adipocyte specific proteins [19,20]. In this study, we provide new evidence that octanoate suppresses lipogenesis, at least in part, by inactivating the key adipocyte transcription factor, peroxisome proliferator-activated receptorγ (PPARγ). Furthermore, our data revealed, for the first time, an involvement of reactive oxygen species (ROS) as a possible intermediate component that might regulate the anti-lipogenic effects.

## Materials and methods

### Materials

3T3-L1 cell line was purchased from American Type Culture Collection (Manassas, VA). The HEK293A cell line was from Invitrogen (Long Island, NY). Cell culture supplies were from Fisher Scientific (Agawa, MA) or Gibco Life Technology (Long Island, NY). TransLucent reporter vector for PPARγ [PPRE(+)-Luc reporter gene containing the PPARγ responsive element (PPRE)] [21] was from Panomics, Inc (Redwood City, CA). *Renilla *luciferase control reporter vector pRL-null and a dual luciferase reporter assay kit system were from Promega (Madison, WI). Recombinant adenovirus encoding glutathione peroxidase and its parental adenovirus Ad-5 were from Genecore of Iowa University (Ames, IA). Troglitazone was from Biomol Inc (Plymouth meeting, PA). Other chemicals, reagents, and solvents were from Sigma (St. Louis, MO), unless noted elsewhere.

### Cell culture

3T3-L1 preadipocytes were grown in Dulbecco's minimum essential medium (DMEM) with 10% calf serum, pencicillin (100 IU), and streptomycin (100 IU). Differentiation was induced on day 2 post confluence using DMEM with 10% fetal bovine serum (FBS), 0.5 mM methylisobutylxanthine, 1 μM dexamethasome, and 170 nM insulin. After 48 h, medium was changed to DMEM plus 10% FBS and 170 nM insulin. Cells were used for incubation with octanoate or other effectors 6–9 days thereafter, at which point >90% of the cells accumulated lipid droplets.

### Lipogenesis

Triglycerides (TAG) synthesis in adipocytes uses both pre-made and *de novo *synthesized fatty acids, with the glycerol backbone comes primarily from glucose-derived glycerol-3-phosphate. In this work, we assess the effects of octanoate on each of these steps using [9,10-^3^H] triolein (1 μCi/ml, 0.5% lipid emulation, measures lipoprotein lipase activity), ^3^H_2_O (25 μCi/ml, measures fatty acid synthase activity), and [U-^3^H] glucose (10 μCi/ml, measures net TAG synthesis) as the substrates. Cells were incubated with labeled substrates individually for 3 h (a linear range of 0 – 5 h was confirmed in preliminary experiments), washed 4 times with warm PBS containing 1% BSA. Cells were then lysed, extracted with organic solvent, and the lipid components were separated by TLC [22]. The TAG fraction was scraped for scintillation counting directly or for methylation reaction as described before [22]. After removal of the methyl acyl esters, the aqueous phase containing the glycerol moiety was used for scintillation counting [22]. Non-specific binding was measured by exposing cells to the same medium but washed immediately (usually <5% of that incorporated into the cellular TAG pool after the 3 h incubation).

### β-oxidation

To measure β-oxidation of octanoate, cells were grown in T25 flask, incubated in serum-free DMEM containing 0.5% BSA overnight, and then treated with exogenous [1-^14^C] octanoate (0.5 mM, 1 μCi/ml) together with the desired effectors for 2 h. The release of ^14^CO_2_was measured as described before [22]. To measure the β-oxidation of oleate, cells grown in 6-well plates were pre-incubated in serum-free DMEM with [9,10-^3^H] oleate (1 μCi/ml, <0.1 μM) overnight. Exogenous oleate was removed by washing the cells with PBS containing 0.5% BSA. Cells were then incubated with or without insulin or L-carnitine for 2 h. The release of ^3^H_2_O into the medium was measured as described [23].

### PPARγ transcriptional activity

HEK293A cells were transfected with PPARγ2 using a recombinant retrovirus encoding a full-length cDNA of mouse PPARγ2 (a gift from Dr. Spiegelman BM, Harvard University) followed by neomycin selection. The 293A-PPARγ2 cells thus generated were then grown in DMEM with 10% FBS to 60% confluence and co-transfected with PPRE(+)-Luc reporter and Rluc reporter vectors using Effectene Transfection Reagent from Qiagen (Valencia, CA). Octanoate or other effectors were added to cell culture 48 h after the transfection. Cells were harvested for a dual luciferase assay using a commercial kit (Promega).

### RNA isolation and real-time RT-PCR analysis

Total RNA was isolated using Trizol method (Invitrogen, Carlsbad, CA) and reverse transcription from mRNA to cDNA was performed as described before [24]. Intron-spanning PCR primers were designed using a web-based program provided by Roche. House keeping gene mouse HPRT was used as the endogenous reference. SYBG-based real-time PCR was conducted in 20 μl reaction mix containing 10 μl PCR enzyme mix (Qiagen), 2 μl cDNA, 3 μl primer mix (final 1.5 μM for each primer), and 5 μl nuclease-free water, using a Rotorgene 3000A system. Amplification parameters consisted of initial enzyme activation at 95°C for 10 min and 45 cycles of three-step PCR (denature 5 s at 95°C, annealing 10 s at 60°C, and extension 20 s at 72°C). The specificity of products generated for each set of primers was examined for each fragment using a melting curve and gel electrophoresis. Reactions were run in triplicates and data calculated as the change in cycle threshold (Ct) for the target gene relative to the Ct for HPRT. To confirm the relationship between Ct values and mRNA levels, primers were calibrated by using serial dilutions of cDNA.

### Cellular ROS

Cellular ROS was measured with a protocol modified from the literature [25]. Briefly, 3T3-L1 adipocytes were incubated with octanoate with or without the antioxidant, N-acetylcysteine (NAC), for 24 h. Dichlorofluorescein diacetate (2 μM, DCFH-DA, Molecular Probe, Eugene, OR), a cell permeable nonfluorescent precursor, was then added to the cells and the incubation was extended for another 30 min. Within the cells, DCFH-DA is hydrolyzed by nonspecific esterases to release DCF, which is readily oxidized by intracellular ROS. The oxidized product emits green fluorescence (ex 488 nm, em 525 nm). At the end of incubation, cells were washed with warm KRB buffer and immediately imaged under a polarizing/fluorescent microscope (Nikon Eclipse TE200). Caution was taken to ensure that cells from different samples were exposed to excitation for identical period of time (30 s) and photographed using the same exposure time (15 s) and receiver gain (1.0) using a Nikon digital camera (original magnification 10x).

### Statistical methods statistics

#### Statistics

Data are shown as means +/- SE. Comparison between two groups of data was made using Student's *t *test. For others, results were analyzed using one-way ANOVA and Duncan's multiple comparison tests. Differences were considered statistically significant when p < 0.05.

## Results

### Octanoate inhibits TAG synthesis and reduces expression of selected lipogenic genes

**Figure 1:**
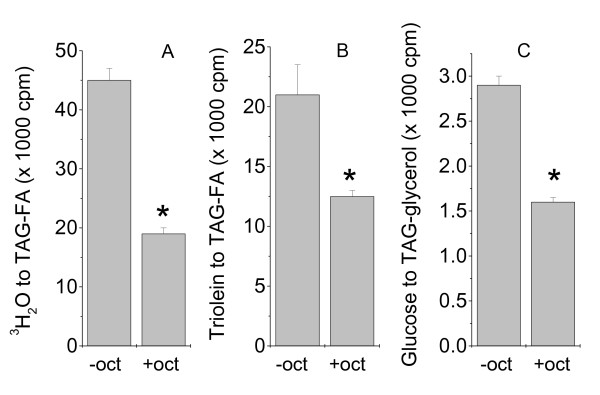
**The effects of octanoate on lipogenesis in adipocytes**. Six days after induction of differentiation, 3T3-L1 adipocytes were incubated with octanoate (1 mM, oct) mixed with DMEM containing 10% FBS and 170 nM insulin for 3 days. Exogenous octanoate were then removed and cells were incubated for 3 h in the same medium with ^3^H_2_O (A), [9,10-^3^H] triolein (B), and [U-^3^H] glucose (C). The incorporation of the corresponding isotope into the cellular TAG-fatty acids (TAG-FA, A&B) and TAG-glycerol (C) were measured. Data are mean +/- SE, n = 3. *p < 0.05 compared to control.

**Figure 2:**
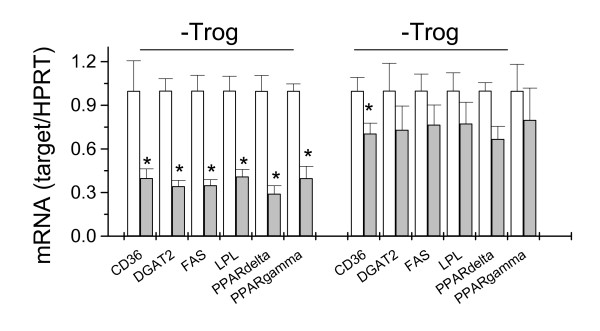
**The effects of octanoate on expression of selected lipogenic genes and the antagonizing effects of troglitazone**. Cells were incubated with octanoate (1 mM) for 24 h with or without troglitazone (5 μM), using DMSO as the vehicle. (Upper panel) The expression of CD36, diacylglycerol acyltransferase 2 (DGAT2), fatty acid synthase (FAS), and lipoprotein lipase (LPL) were analyzed by RT-qPCR using house keeping gene HPRT as the endogenous reference. The results were normalized to control cells. Results are mean +/- SE, n = 3, *p < 0.05.

In this study, TAG synthesis was measured by isotope-tracing method using substrates at different stages of the biosynthesis cascade. As shown in Figure 1, pre-incubation with octanoate for 3 days substantially inhibited the *de novo *fatty acid synthesis (Fig. 1A), the incorporation of exogenous fatty acids into TAG (Fig. 1B), and the net synthesis of intracellular TAG (Fig. 1C). These results indicated that octanoate induced a comprehensive inhibition of lipogenesis. Since measurement was done in the absence of octanoate, the inhibitory effect was likely sustained through modulation of gene expression. To test this possibility, we performed quantitative real-time PCR analysis for selected lipogenic genes. As shown in Figure 2, octanoate induced a large decrease in the expression of key enzymes involved in fatty acid uptake and triglyceride synthesis. These included lipoprotein lipase (LPL), fatty acid synthase (FAS), and diacylglycerol acyltransferase 2 (DGAT2). The reduction in LPL and FAS correlates with the specific reduction in fatty acid uptake from exogenous triolein (Fig. 1B) and de novo fatty acid synthesis from H_2_O (Fig. 1A), respectively. A reduction in DGAT2 might contribute to reduction of overall triglyceride synthesis (Fig. 1A–C). Besides, octanoate also inhibited the expression of CD36, a protein that has been shown to be required for efficient lipid storage [26]. Among the targets tested, CD36 and LPL have been demonstrated as PPARγ target genes [27,28]. As for FAS and DGAT2, although with no defined PPRE in their promoters, both are drastically induced during preadipocyte differentiation, a process that is tightly controlled by the activation of PPARγ. Hence, these two genes can be considered as indirect downstream targets of PPARγ. It is not surprising that we detected a large decrease in mRNA of PPARγ in association with the changes in the aforementioned lipogenic genes (Fig. 2). In parallel, we also detected a similar decrease in the expression of PPARδ (Fig. 2). Co-incubation with a synthetic PPARγ agonist, troglitazone, largely restored the expression of the lipogenic genes as well as PPARγ amd PPARδ (Fig. 2). Troglitazone also caused a 1.5 fold increase in TAG synthesis and diminished the inhibitory effect of octanoate ([19] and data not shown).

### β-oxidation of octanoate is largely independent of CPT-I

**Figure 3:**
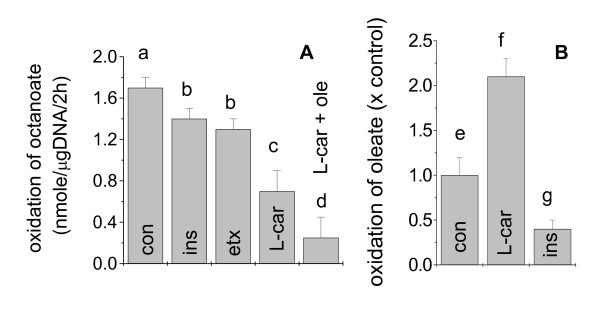
**The regulation of β-oxidation of octanoate (A) and oleate (B) in adipocytes**. Cells were serum-starved overnight in DMEM containing 0.5% BSA. (A) Cells were then incubated in DMEM for 2 h with exogenous octanoate [0.5 mM, 1 μCi/ml)] alone or together with insulin (170 nM, ins), etomoxir (30 μM, etx), L-carnitine (5 mM, L-car), or L-carnitine plus oleate (0.5 mM, L-car + ole). The release of ^14^CO_2 _was measured. (B) Cells were pre-incubated with [9,10-^3^H] oleate overnight. After washing off the exogenous oleate, cells were incubated with L-carnitine (5 mM) or insulin (170 nM) in DMEM for 2 h. The release of ^3^H_2_O into the medium was measured. Results are mean +/- SE. Values indicated with different letters are significantly different (p < 0.05).

Results above suggest that sustained anti-liogenic effect of octanoate is correlated with reduced expression of lipogenic genes, and the effect was reversible by co-treatment with a PPARγ synthetic ligand. This distinguishes octanoate from common fatty acids that have been shown to activate PPARγ [29-36]. One of the unique properties of octanoate is that it might be activated within the mitochondria [12] and enters the β-oxidation pathway independent of CPT-I [37]. This allows octanoate to be β-oxidized in the presence of glucose and insulin, conditions under which long-chain fatty acids are primarily channeled to esterification. This prediction, however, has not been firmly established in adipocytes. Peculiarly, several isoforms of medium chain acyl CoA synthetase have been recently identified and cloned [38-40], with none expressed to an appreciable level in adipocytes [41]. Because β-oxidation is linked to ROS generation [25,42-44], the potential molecular signals for regulation of lipogenesis, it is important to test whether β-oxidation of octanoate is truly independent of CPT-I in adipocytes. For this purpose, we measured the generation of ^14^CO_2 _from octanoate and tested its response to insulin, L-carnitine, oleate, and etomoxir, factors that modulate CPT-I via different mechanisms. As shown in Figure 3A, β-oxidation of octanoate was slightly inhibited (~18%) by insulin, a hormone that promotes the generation of the natural inhibitor of CPT-I [37], and Etomoxir, a pharmaceutical inhibitor of CPT-I. On the other hand, L-carnitine, an activator of CPT-I, caused a ~60% inhibition of octanoate oxidation. A combination of L-carnitine and exogenous oleate further enhanced the inhibition (> 85%). In contrast, β-oxidation of oleate was increased by L-carnitine more than 2 fold but inhibited by insulin by about 60% (Fig. 3B), consistent with the literature [37]. These results indicate that in adipocytes, octanoate was mainly oxidized independent of CPT-I (> 80%). A small fraction (< 20%), that was sensitive to insulin and etomoxir, might be activated in the cytosol and hence depend on CPT-I to enter the mitochondria. The observation that L-carnitine inhibited, rather than promoted, β-oxidation of octanoate suggests that activation of CPT-I largely increased the transport of endogenous fatty acids into the β-oxidation pathway which compete with octanoate for the enzymes downstream from CPT-1. This competition was further enhanced in the presence of added oleate.

### Octanoate-induced ROS generation and inhibition of PPARγ activity: effects of N-acetylcysteine

**Figure 4:**
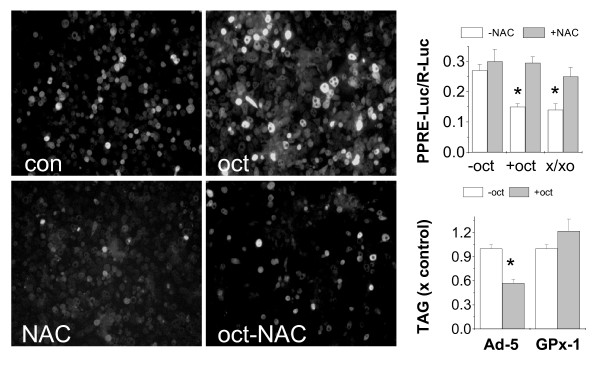
**The effects of octanoate on the generation of reactive oxygen species (ROS, A), PPARγ transcription activity (B) and TAG synthesis (C), and the effects of antioxidants**. For (A), 3T3-L1 adipocytes were incubated with octanoate (1 mM) for 24 h with or without N-acetylcysteine (NAC, 20 mM) followed with the measurement of ROS by the DCF assay. Results are representative of at least three independent experiments. For (B), 293A-PPARγ2 cells were co-transfected with the plasmid DNA vector encoding a PPRE(+)-Luc reporter gene or the Rluc control reporter gene. After 24 h, cells incubated with octanoate (1 mM) or a mixture of xanthine/xanthine oxidase (200 μM/30 μU/ml). Cells were harvested after 24 h and analyzed using a dual luciferase assay. For (C), 3T3-L1 adipocytes were infected with recombinant adenovirus encoding GPx1 or its parental virus Ad-5. After 24 h, Cells were incubated with octanoate (1 mM) for another 3 days. TAG synthesis from [U-^3^H] glucose was measured as described in Figure 1. For (B&C), Results are mean +/- SE, n = 3, *p < 0.05 compared to control.

A common consequence of fatty acid oxidation, as compared to that of glucose, is the generation of NADH and FADH2. This increases electron flow through the mitochondrial electron transport chain and increases electron leak to produce ROS [45,46]. Because ROS has been shown to activate the stress-responsive protein kinases that lead to the inhibition of PPARγ [47-49], we hypothesize that ROS might be an important mediator for the anti-lipogenic effects of octanoate. To test this possibility, we first measured the ROS intensity in adipocytes treated with octanoate. As shown in Figure 4A, incubation with octanoate for 24 h significantly increased the intracellular ROS intensity, which was blocked by co-treatment with N-acetylcysteine. Secondly, we measured the effects of octanoate on PPARγ transcriptional activity using a PPRE(+)-Luc reporter gene assay. As shown in Figure 4B, pre-incubation with octanoate for 24 h inhibited PPARγ transcription activity by about 50%, and this inhibition was blocked by a co-treatment with N-acetylcysteine. As a positive control, we show that exogenous ROS, generated by a mixture of xanthine and xanthine oxidase, also inhibited PPARγ transcriptional activity, an effect blocked by N-acetylcysteine. Finally, we transfected mature adipocytes with the adenovirus encoding glutathione peroxidase 1 (GPx-1), a cytosolic isoform of GPx which detoxifies ROS [47]. As shown in Figure 4C, octanoate inhibited TAG synthesis by about 40% in adipocytes transfected with the parent adenovirus Ad-5. This inhibition was offset by over-expression of GPx-1.

## Discussion

Fatty acid oxidation is normally activated only under fasting conditions when circulating levels of insulin and glucose are low. Conversely, lipogenesis is down-regulated by fasting. The mechanistic link between these two events, however, has not been established. In this work, we provided the first evidence that medium-chain octanoate can be β-oxidized in adipocytes independent of CPT-I regulation. Hence, supplement of octanoate maintains active β-oxidation in the presence of insulin and glucose. This is correlated with inhibition of lipogenesis and reduction of lipogenic gene expression. In other words, octanoate induces a metabolic state in adipocytes mimicking a fasting condition without actual hormone/nutrient deprivation. Our results also demonstrated that ROS might be involved as a mediator for octanoate in lowering PPARγ activity, the master control of lipogenic gene expression.

As extensively reviewed previously, PPARγ is a prototypical member of the nuclear receptor superfamily which integrates the control of energy, lipid and glucose, homerostasis [50-54]. PPARγ binds a variety of small lipophilic compounds derived from metabolism and nutrition. These ligands, in turn, determine cofactor recruitment and regulate the transcription of a variety of metabolic genes. Recent literature highlights the development of partial agonists of PPARγ to block adipogenesis and reduce fat mass development [54-59]. In one of our previous studies, we proposed that octanoate might act as a partial agonist for PPARγ because it can potentially bind to PPARγ as does the long-chain fatty acids [29,60], hence competitively blocking the binding of the latter or other endogenous ligands. This model was supported, but not proved, by the findings that the anti-adipogenic [19] and anti-lipogenic (this work) effects of octanoate was efficiently blocked by selected synthetic PPARγ agonists.

The current findings that octanoate induced ROS generation in adipocytes suggest that octanoate might also modulate PPARγ activity indirectly via the ROS signaling pathways. It has been well established that ROS activates the stress-responsive protein kinases [61,62], which either directly or indirectly inhibit PPARγ activity [47-49,62-67]. In our preliminary studies, we found that octanoate also induced sustained activation of Erk1/2 and JNK/SAPK (data not shown). How these kinase pathways are involved in the regulation of PPARγ activity and lipogenesis in our cell system and, more importantly, in primary adipocytes, are currently under investigation.

Inhibition of adipocyte lipogenesis can be a useful tool for the prevention of obesity. In this regard, our studies contribute to the scientific basis for the application of MCT in dietary weight management. On the other hand, a complete inhibition of fat mass growth is disastrous since adipocytes play important roles in physiological functions of mammals. Compared to the pharmaceutical inhibitors of lipogenesis [68,69], the effects of octanoate can be considered as moderate and yet might be more desirable for physiological regulation of body fat mass without adversely affecting normal fat tissue functions. According to recent surveys, a majority of the middle age population is moderately over-weighed (BMI 23–27), and a slight increase in BMI in this range is associated with a greater risk for metabolic syndrome [70,71]. It will be of important social and economical values if MCT can be used for body weight regulation in this sub-population, as demonstrated by a recent clinical trial [5].

## Conclusion

This study demonstrated that octanoate had a direct inhibitory effect on fat storage in adipocytes under conditions that normally favor lipogenesis. This was related to its unique β-oxidation mechanism which links to elevated cellular ROS levels and subsequent inactivation of PPARγ. The exact mechanism by which PPARγ is inactivated, in particular, how ROS is involved in this process, still remains to be elucidated. Furthermore, ROS is known to have diverse and complex molecular targets, which might directly or indirectly influence the activities of additional adipocyte transcription factors or modify selected lipogenic proteins [44,71]. Elucidation of these mechanisms will be helpful for the application of MCT for dietary intervention to prevent obesity and may reveal possible pharmaceutical targets to modulate fat metabolism.

## Competing interests

The author(s) declare that they have no competing interests.

## Authors' contributions

WG performed the experiments for fatty acid oxidation, fatty acid and triglyceride synthesis and luciferase activity measurements, real time PCR analysis, as well as prepared the manuscript. WX performed the experiments for ROS analysis.

## References

[B1] Bray GA, Lee M, Bray TL (1980). Weight gain of rats fed medium-chain triglycerides is less than rats fed long-chain triglycerides. Int J Obes.

[B2] Hashim SA, Tantibhedyangkul P (1987). Medium chain triglyceride in early life: effects on growth of adipose tissue. Lipids.

[B3] Papamandjaris AA, White MD, Raeini-Sarjaz M, Jones PJ (2000). Endogenous fat oxidation during medium chain versus long chain triglyceride feeding in healthy women. Int J Obes Relat Metab Disord.

[B4] Tsuji H, Kasai M, Takeuchi H, Nakamura M, Okazaki M, Kondo K (2001). Dietary medium-chain triacylglycerols suppress accumulation of body fat in a double-blind, controlled trial in healthy men and women. J Nutr.

[B5] Nosaka N, Maki H, Suzuki Y, Haruna H, Ohara A, Kasai M, Tsuji H, Aoyama T, Okazaki M, Igarashi O, Kondo K (2003). Effects of margarine containing medium-chain triacylglycerols on body fat reduction in humans. J Atheroscler Thromb.

[B6] Han J, Hamilton JA, Kirkland JL, Corkey BE, Guo W (2003). Medium-chain oil reduces fat mass and down-regulates expression of adipogenic genes in rats. Obes Res.

[B7] St-Onge MP, Ross R, Parsons WD, Jones PJ (2003). Medium-chain triglycerides increase energy expenditure and decrease adiposity in overweight men. Obes Res.

[B8] St-Onge MP, Jones PJ (2003). Greater rise in fat oxidation with medium-chain triglyceride consumption relative to long-chain triglyceride is associated with lower initial body weight and greater loss of subcutaneous adipose tissue. Int J Obes Relat Metab Disord.

[B9] Bourque C, St-Onge MP, Papamandjaris AA, Cohn JS, Jones PJ (2003). Consumption of an oil composed of medium chain triacyglycerols, phytosterols, and N-3 fatty acids improves cardiovascular risk profile in overweight women. Metabolism.

[B10] St-Onge MP, Bourque C, Jones PJ, Ross R, Parsons WE (2003). Medium- versus long-chain triglycerides for 27 days increases fat oxidation and energy expenditure without resulting in changes in body composition in overweight women. Int J Obes Relat Metab Disord.

[B11] Bach AC, Ingenbleek Y, Frey A (1996). The usefulness of dietary medium-chain triglycerides in body weight control: fact or fancy?. J Lipid Res.

[B12] Aas M (1971). Organ and subcellular distribution of fatty acid activating enzymes in the rat. Biochim Biophys Acta.

[B13] Wiley JH, Leveille GA (1973). Metabolic consequences of dietary medium-chain triglycerides in the rat. J Nutr.

[B14] Lavau MM, Hashim SA (1978). Effect of medium chain triglyceride on lipogenesis and body fat in the rat. J Nutr.

[B15] Hill JO, Peters JC, Lin D, Yakubu F, Greene H, Swift L (1993). Lipid accumulation and body fat distribution is influenced by type of dietary fat fed to rats. Int J Obes Relat Metab Disord.

[B16] Kinkela T, Chanussot F, Bach A, Max JP, Schirardin H, Debry G (1983). Effects of diets containing medium-chain and long-chain triacylglycerols in the genetically obese Zucker fa/fa rat. Composition of fatty acids and triacylglycerols of the liver and adipose tissues. Ann Nutr Metab.

[B17] Sarda P, Lepage G, Roy CC, Chessex P (1987). Storage of medium-chain triglycerides in adipose tissue of orally fed infants. Am J Clin Nutr.

[B18] Guo W, Lei T, Wang T, Corkey BE, Han J (2003). Octanoate inhibits triglyceride synthesis in 3T3-L1 and human adipocytes. J Nutr.

[B19] Han J, Farmer SR, Kirkland JL, Corkey BE, Yoon R, Pirtskhalava T, Ido Y, Guo W (2002). Octanoate attenuates adipogenesis in 3T3-L1 preadipocytes. J Nutr.

[B20] Nakajima I, Muroya S, Chikuni K (2003). Growth arrest by octanoate is required for porcine preadipocyte differentiation. Biochem Biophys Res Commun.

[B21] Graves RA, Tontonoz P, Spiegelman BM (1992). Analysis of a tissue-specific enhancer: ARF6 regulates adipogenic gene expression. Mol Cell Biol.

[B22] Guo W, Choi JK, Kirkland JL, Corkey BE, Hamilton JA (2000). Esterification of free fatty acids in adipocytes: a comparison between octanoate and oleate. Biochem J.

[B23] Wang T, Zang Y, Ling W, Corkey BE, Guo W (2003). Metabolic partitioning of endogenous fatty acid in adipocytes. Obes Res.

[B24] Guo WLT, Wang T, Corkey BE, Han J (2003). Octanoate inhibits triglyceride synthesis in 3T3-L1 and human adipocytes. J Nutr.

[B25] Talior I, Yarkoni M, Bashan N, Eldar-Finkelman H (2003). Increased glucose uptake promotes oxidative stress and PKC-delta activation in adipocytes of obese, insulin-resistant mice. Am J Physiol Endocrinol Metab.

[B26] Hajri T, Han XX, Bonen A, Abumrad NA (2002). Defective fatty acid uptake modulates insulin responsiveness and metabolic responses to diet in CD36-null mice. J Clin Invest.

[B27] Schoonjans K, Peinado-Onsurbe J, Lefebvre AM, Heyman RA, Briggs M, Deeb S, Staels B, Auwerx J PPARalpha and PPARgamma activators direct a distinct tissue-specific transcriptional response via a PPRE in the lipoprotein lipase gene. EMBO J.

[B28] Sato O, Kuriki C, Fukui Y, Motojima K (2002). Dual promoter structure of mouse and human fatty acid translocase/CD36 genes and unique transcriptional activation by peroxisome proliferator-activated receptor alpha and gamma ligands. J Biol Chem.

[B29] Kliewer SA, Sundseth SS, Jones SA, Brown PJ, Wisely GB, Koble CS, Devchand P, Wahli W, Willson TM, Lenhard JM, Lehmann JM (1997). Fatty acids and eicosanoids regulate gene expression through direct interactions with peroxisome proliferator-activated receptors alpha and gamma. Proc Natl Acad Sci USA.

[B30] Shillabeer G, Lau DC (1994). Regulation of new fat cell formation in rats: the role of dietary fats. J Lipid Res.

[B31] Shillabeer G, Forden JM, Lau DC (1989). Induction of preadipocyte differentiation by mature fat cells in the rat. J Clin Invest.

[B32] Brandes R, Arad R, Bar-Tana J (1995). Inducers of adipose conversion activate transcription promoted by a peroxisome proliferators response element in 3T3-L1 cells. Biochem Pharmacol.

[B33] Ibrahimi A, Teboul L, Gaillard D, Amri EZ, Ailhaud G, Young P, Cawthorne MA, Grimaldi PA (1994). Evidence for a common mechanism of action for fatty acids and thiazolidinedione antidiabetic agents on gene expression in preadipose cells. Mol Pharmacol.

[B34] Ailhaud G, Amri EZ, Grimaldi PA (1996). Fatty acids and expression of lipid-related genes in adipose cells. Proc Nutr Soc.

[B35] Ailhaud G, Amri EZ, Grimaldi PA (1995). Fatty acids and adipose cell differentiation. Prostaglandins Leukot Essent Fatty Acids.

[B36] Amri EZ, Bertrand B, Ailhaud G, Grimaldi P (1991). Regulation of adipose cell differentiation. I. Fatty acids are inducers of the aP2 gene expression. J Lipid Res.

[B37] McGarry JD, Sen A, Esser V, Woeltje KF, Weis B, Foster DW (1991). New insights into the mitochondrial carnitine palmitoyltransferase enzyme system. Biochimie.

[B38] Fujino T, Takei YA, Sone H, Ioka RX, Kamataki A, Magoori K, Takahashi S, Sakai J, Yamamoto TT (2001). Molecular identification and characterization of two medium-chain acyl-CoA synthetases, MACS1 and the Sa gene product. J Biol Chem.

[B39] Vessey DA, Lau E, Kelley M, Warren RS (2003). Isolation, sequencing, and expression of a cDNA for the HXM-A form of xenobiotic/medium-chain fatty acid:CoA ligase from human liver mitochondria. J Biochem Mol Toxicol.

[B40] Oka Y, Kobayakawa K, Nishizumi H, Miyamichi K, Hirose S, Tsuboi A, Sakano H (2003). O-MACS, a novel member of the medium-chain acyl-CoA synthetase family, specifically expressed in the olfactory epithelium in a zone-specific manner. Eur J Biochem.

[B41] Lei T, Xie W, Watkins PA, Guo W (2003). Activation of medium-chain fatty acids in 3T3-L1 adipocytes and mouse adipose tissue. Obes Res.

[B42] Hickson-Bick DL, Sparagna GC, Buja LM, McMillin JB (2002). Palmitate-induced apoptosis in neonatal cardiomyocytes is not dependent on the generation of ROS. Am J Physiol Heart Circ Physiol.

[B43] Yamagishi SI, Edelstein D, Du XL, Kaneda Y, Guzman M, Brownlee M (2001). Leptin induces mitochondrial superoxide production and monocyte chemoattractant protein-1 expression in aortic endothelial cells by increasing fatty acid oxidation via protein kinase A. J Biol Chem.

[B44] Yamagishi S, Okamoto T, Amano S, Inagaki Y, Koga K, Koga M, Choei H, Sasaki N, Kikuchi S, Takeuchi M, Makita Z (2002). Palmitate-induced apoptosis of microvascular endothelial cells and pericytes. Mol Med.

[B45] Turrens JF, Alexandre A, Lehninger AL (1985). Ubisemiquinone is the electron donor for superoxide formation by complex III of heart mitochondria. Arch Biochem Biophys.

[B46] Young TA, Cunningham CC, Bailey SM (2002). Reactive oxygen species production by the mitochondrial respiratory chain in isolated rat hepatocytes and liver mitochondria: studies using myxothiazol. Arch Biochem Biophys.

[B47] Hu E, Kim JB, Sarraf P, Spiegelman BM (1996). Inhibition of adipogenesis through MAP kinase-mediated phosphorylation of PPARgamma. Science.

[B48] Yang W, Hong YH, Shen XQ, Frankowski C, Camp HS, Leff T (2001). Regulation of transcription by AMP-activated protein kinase: phosphorylation of p300 blocks its interaction with nuclear receptors. J Biol Chem.

[B49] Camp HS, Tafuri SR, Leff T (1999). c-Jun N-terminal kinase phosphorylates peroxisome proliferator-activated receptor-gamma1 and negatively regulates its transcriptional activity. Endocrinology.

[B50] Debril MB, Renaud JP, Fajas L, Auwerx J (2001). The pleiotropic functions of peroxisome proliferator-activated receptor gamma. J Mol Med.

[B51] Rosen ED, Spiegelman BM (2001). PPARgamma: a nuclear regulator of metabolism, differentiation, and cell growth. J Biol Chem.

[B52] Hihi AK, Michalik L, Wahli W (2002). PPARs: transcriptional effectors of fatty acids and their derivatives. Cell Mol Life Sci.

[B53] Sewter C, Vidal-Puig A (2002). PPARgamma and the thiazolidinediones: molecular basis for a treatment of 'Syndrome X'?. Diabetes Obes Metab.

[B54] Knouff C, Auwerx J (2004). Peroxisome proliferator-activated receptor-gamma calls for activation in moderation: lessons from genetics and pharmacology. Endocr Rev.

[B55] Oberfield JL, Collins JL, Holmes CP, Goreham DM, Cooper JP, Cobb JE, Lenhard JM, Hull-Ryde EA, Mohr CP, Blanchard SG, Parks DJ, Moore LB, Lehmann JM, Plunket K, Miller AB, Milburn MV, Kliewer SA, Willson TM (1999). A peroxisome proliferator-activated receptor gamma ligand inhibits adipocyte differentiation. Proc Natl Acad Sci U S A.

[B56] Mukherjee R, Hoener PA, Jow L, Bilakovics J, Klausing K, Mais DE, Faulkner A, Croston GE, Paterniti JR (2000). A selective peroxisome proliferator-activated receptor-gamma (PPARgamma) modulator blocks adipocyte differentiation but stimulates glucose uptake in 3T3-L1 adipocytes. Mol Endocrinol.

[B57] Camp HS, Chaudhry A, Leff T (2001). A novel potent antagonist of peroxisome proliferator-activated receptor gamma blocks adipocyte differentiation but does not revert the phenotype of terminally differentiated adipocytes. Endocrinology.

[B58] Lee G, Elwood F, McNally J, Weiszmann J, Lindstrom M, Amaral K, Nakamura M, Miao S, Cao P, Learned RM, Chen JL, Li Y (0907). T007 a selective ligand for peroxisome proliferator-activated receptor gamma, functions as an antagonist of biochemical and cellular activities. J Biol Chem.

[B59] Leesnitzer LM, Parks DJ, Bledsoe RK, Cobb JE, Collins JL, Consler TG, Davis RG, Hull-Ryde EA, Lenhard JM, Patel L, Plunket KD, Shenk JL, Stimmel JB, Therapontos C, Willson TM, Blanchard SG (2002). Functional consequences of cysteine modification in the ligand binding sites of peroxisome proliferator activated receptors by GW9662. Biochemistry.

[B60] Xu HE, Lambert MH, Montana VG, Parks DJ, Blanchard SG, Brown PJ, Sternbach DD, Lehmann JM, Wisely GB, Willson TM, Kliewer SA, Milburn MV (1999). Molecular recognition of fatty acids by peroxisome proliferator-activated receptors. Mol Cell.

[B61] Choi SL, Kim SJ, Lee KT, Kim J, Mu J, Birnbaum MJ, Soo Kim S, Ha J (2001). The regulation of AMP-activated protein kinase by H(2)O(2). Biochem Biophys Res Commun.

[B62] Souza SC, Palmer HJ, Kang YH, Yamamoto MT, Muliro KV, Paulson KE, Greenberg AS (2003). TNF-alpha induction of lipolysis is mediated through activation of the extracellular signal related kinase pathway in 3T3-L1 adipocytes. J Cell Biochem.

[B63] Floyd ZE, Stephens JM (2002). Interferon-gamma-mediated activation and ubiquitin-proteasome-dependent degradation of PPARgamma in adipocytes. J Biol Chem.

[B64] Huang WC, Chio CC, Chi KH, Wu HM, Lin WW (2002). Superoxide anion-dependent Raf/MEK/ERK activation by peroxisome proliferator activated receptor gamma agonists 15-deoxy-delta(12,14)-prostaglandin J(2), ciglitazone, and GW1929. Exp Cell Res.

[B65] Hedvat M, Jain A, Carson DA, Leoni LM, Huang G, Holden S, Lu D, Corr M, Fox W, Agus DB (2004). Inhibition of HER-kinase activation prevents ERK-mediated degradation of PPARgamma. Cancer Cell.

[B66] Tanabe Y, Nakayama K (2004). Mechanical stretching inhibits adipocyte differentiation of 3T3-L1 cells: the molecular mechanism and pharmacological regulation. Nippon Yakurigaku Zasshi.

[B67] Tanabe Y, Koga M, Saito M, Matsunaga Y, Nakayama K (2004). Inhibition of adipocyte differentiation by mechanical stretching through ERK-mediated downregulation of PPARgamma2. J Cell Sci.

[B68] Goransson O, Ryden M, Nilsson R, Arner P, Degerman E (2004). Dimethylaminopurine inhibits metabolic effects of insulin in primary adipocytes. In J Nutr Biochem.

[B69] Thupari JN, Landree LE, Ronnett GV, Kuhajda FP (2002). C75 increases peripheral energy utilization and fatty acid oxidation in diet-induced obesity. Proc Natl Acad Sci U S A.

[B70] St-Onge MP (2005). Relationship between body composition changes and changes in physical function and metabolic risk factors in aging. Curr Opin Clin Nutr Metab Care.

[B71] St-Onge MP, Janssen I, Heymsfield SB (2004). Metabolic syndrome in normal-weight Americans: new definition of the metabolically obese, normal-weight individual. Diabetes Care.

